# Comparison of the efficacy and safety of rosuvastatin 10 mg and atorvastatin 20 mg in high-risk patients with hypercholesterolemia – Prospective study to evaluate the Use of Low doses of the Statins Atorvastatin and Rosuvastatin (PULSAR)

**DOI:** 10.1186/1745-6215-7-35

**Published:** 2006-12-21

**Authors:** Michael B Clearfield, John Amerena, Jean-Pierre Bassand, Hugo R Hernández García, Sam S Miller, Froukje FM Sosef, Michael K Palmer, Brian S Bryzinski

**Affiliations:** 1 University of North Texas Health Science Center, Fort Worth, TX, USA; 2 Department of Clinical and Biomedical Sciences, University of Melbourne, Melbourne, Australia; 3 University Hospital Besançon, Besançon, France; 4 Hospital de Especialidades, Centro Medico de Occidente, Guadalajara, Mexico; 5 SAM Clinical Research Center, San Antonio, TX, USA; 6 AstraZeneca, Alderley Park, Macclesfield, Cheshire, UK; 7 AstraZeneca, Wilmington, DE, USA; 8 Present address: College of Osteopathic Medicine, Touro University, 1310 Johnson Lane, Vallejo, CA 94592, USA

## Abstract

**Background:**

Many patients at high risk of cardiovascular disease do not achieve recommended low-density lipoprotein cholesterol (LDL-C) goals. This study compared the efficacy and safety of low doses of rosuvastatin (10 mg) and atorvastatin (20 mg) in high-risk patients with hypercholesterolemia.

**Methods:**

A total of 996 patients with hypercholesterolemia (LDL-C ≥ 3.4 and < 5.7 mmol/L [130 and 220 mg/dL]) and coronary heart disease (CHD), atherosclerosis, or a CHD-risk equivalent were randomized to once-daily rosuvastatin 10 mg or atorvastatin 20 mg. The primary endpoint was the percentage change from baseline in LDL-C levels at 6 weeks. Secondary endpoints included LDL-C goal achievement (National Cholesterol Education Program Adult Treatment Panel III [NCEP ATP III] goal < 100 mg/dL; 2003 European goal < 2.5 mmol/L for patients with atherosclerotic disease, type 2 diabetes, or at high risk of cardiovascular events, as assessed by a Systematic COronary Risk Evaluation (SCORE) risk ≥ 5% or 3.0 mmol/L for all other patients), changes in other lipids and lipoproteins, cost-effectiveness, and safety.

**Results:**

Rosuvastatin 10 mg reduced LDL-C levels significantly more than atorvastatin 20 mg at week 6 (44.6% vs. 42.7%, p < 0.05). Significantly more patients achieved NCEP ATP III and 2003 European LDL-C goals with rosuvastatin 10 mg compared with atorvastatin 20 mg (68.8% vs. 62.5%, p < 0.05; 68.0% vs. 63.3%, p < 0.05, respectively). High-density lipoprotein cholesterol was increased significantly with rosuvastatin 10 mg versus atorvastatin 20 mg (6.4% vs. 3.1%, p < 0.001). Lipid ratios and levels of apolipoprotein A-I also improved more with rosuvastatin 10 mg than with atorvastatin 20 mg. The use of rosuvastatin 10 mg was also cost-effective compared with atorvastatin 20 mg in both a US and a UK setting. Both treatments were well tolerated, with a similar incidence of adverse events (rosuvastatin 10 mg, 27.5%; atorvastatin 20 mg, 26.1%). No cases of rhabdomyolysis, liver, or renal insufficiency were recorded.

**Conclusion:**

In high-risk patients with hypercholesterolemia, rosuvastatin 10 mg was more efficacious than atorvastatin 20 mg at reducing LDL-C, enabling LDL-C goal achievement and improving other lipid parameters. Both treatments were well tolerated.

## Background

There is a wealth of evidence suggesting that lowering low-density lipoprotein cholesterol (LDL-C) reduces the risk of cardiovascular disease (CVD) [1-4]. Both European and US guidelines for CVD prevention recommend the use of 3-hydroxy-3-methylgluatryl coenzyme A reductase inhibitors (statins) as first-line therapy for dyslipidemia and specify target LDL-C levels [5,6]. More recently, a National Cholesterol Education Program (NCEP) report has proposed to lower target levels to even more aggressive LDL-C goals for very high-risk patients [7].

Despite the proven benefits of LDL-C reduction, lipid management is suboptimal and many patients fail to achieve recommended LDL-C goals [8-10]. The most likely reasons for this are the use of agents with a poor efficacy for LDL-C lowering and suboptimal dose titration. In high-risk patients with elevated LDL-C, goal attainment is particularly poor since treatment with higher doses of statins is often necessary to achieve their target LDL-C levels. Furthermore, these patients are set more aggressive LDL-C goals, which are consequently harder to achieve. The most effective statin at the lowest dose would represent a simple, effective treatment strategy, enabling more patients to achieve goals without the need for dose titration.

Rosuvastatin, at a dose of 10 mg, has demonstrated high efficacy for LDL-C lowering, enabling patients with hypercholesterolemia to achieve their lipid goals [11,12]. In addition, rosuvastatin has beneficial effects on other components of the lipid profile, including high-density lipoprotein cholesterol (HDL-C) [11,13,14], which is a major, independent risk factor for CVD[5,6]. Safety data from several large-scale clinical and pharmacoepidemiologic studies has shown that the safety of rosuvastatin 10–40 mg was similar to that observed for the other statins studied and that rosuvastatin demonstrated a favorable benefit-risk profile across this dose range [15-18]. Results from a recent study by the National Lipid Association (NLA) also support these findings [19].

The aim of the PULSAR (**P**rospective study to evaluate the **U**se of **L**ow doses of the **S**tatins **A**torvastatin and **R**osuvastatin, 4522IL/0102) study was to compare the efficacy and safety of rosuvastatin 10 mg/day and atorvastatin 20 mg/day for 6 weeks in high-risk patients with hypercholesterolemia and known coronary heart disease (CHD), atherosclerosis or a CHD-risk equivalent. The doses chosen for the study were generally recommended start doses of rosuvastatin (10 mg) and atorvastatin (20 mg). PULSAR is the first, prospective, large-scale, multinational study designed to compare low doses of rosuvastatin and atorvastatin for their LDL-C-lowering efficacy in high-risk patients.

## Methods

### Trial design

This was a 6-week, open-label, randomized, parallel-group study conducted in 121 centers in Australia, Finland, France, Italy, Mexico, the Netherlands, and the USA between November 2003 and August 2004. Patients were required to discontinue all lipid-lowering therapy before entering a 6-week dietary lead-in period, during which they followed the NCEP Adult Treatment Panel (ATP) III Therapeutic Lifestyle Changes diet [6]. Patients were then randomized 1:1 to once-daily oral treatment with rosuvastatin 10 mg or atorvastatin 20 mg for 6 weeks. Randomization was conducted by telephone using an interactive voice recognition system.

The study was performed in accordance with the ethical principles in the Declaration of Helsinki and was consistent with International Conference of Harmonisation Good Clinical Practice guidelines and applicable regulatory requirements. It was approved by the local institutional review boards of all the participating centers, and all patients gave written, informed consent before enrollment.

### Patients

Men and women aged ≥ 18 years of age with hypercholesterolemia and either a history of CHD, clinical evidence of atherosclerosis or a CHD-risk equivalent (other clinical form of atherosclerotic disease [peripheral arterial disease, abdominal aortic aneurysm or symptomatic carotid artery disease (transient ischemic attacks, stroke of carotid origin, or > 50% obstruction of a carotid artery)], diabetes mellitus or ≥ 2 risk factors that confer a 10-year CHD-risk score > 20%) were eligible for randomization to the study if the mean of their 2 most recent fasting LDL-C levels was ≥ 3.4 and < 5.7 mmol/L (130 and 220 mg/dL), and the two measurements were within 15% of each other. Eligible patients were also required to have fasting triglycerides (TG) < 4.5 mmol/L (400 mg/dL).

Exclusion criteria included: history of statin-induced myopathy or a serious hypersensitivity reaction to statins; patients considered to be unstable (event within 8–12 weeks) after a myocardial infarction (MI), unstable angina, myocardial revascularization (percutaneous transluminal coronary angioplasty, coronary artery bypass graft surgery, or another revascularization procedure) or a transient ischemic attack or stroke; patients awaiting a planned myocardial revascularization; severe congestive heart failure (New York Heart Association class IIIb or IV); history of malignancy; history of known homozygous familial hypercholesterolemia; current active liver disease (alanine aminotransferase [ALT] > 2 × upper limit of normal [ULN]); unexplained creatine kinase (CK) ≥ 3 × ULN; serum creatinine > 176 μmol/L (2.0 mg/dL); uncontrolled hypothyroidism (thyroid-stimulating hormone > 1.5 × ULN); a history of alcohol or drug abuse within the last 5 years, and initiation of hormone-replacement therapy or oral contraceptives within 3 months before enrollment. In addition, women who were pregnant, breast-feeding or of child-bearing potential and not using a reliable form of contraception were excluded.

Disallowed concomitant medications included erythromycin, fluconazole, ketoconazole, and itraconazole, lipid-modifying drugs, such as niacin, and certain immunosuppressants, such as cyclosporine. Other restrictions during the study included fasting for 8 hours before each visit and avoiding alcohol or cigarettes on the morning of each visit.

### Objectives

The primary endpoint was the percentage change from baseline (at randomization, week 0) in LDL-C levels after 6 weeks of treatment. Secondary efficacy endpoints included: percentage of patients achieving LDL-C goals (NCEP ATP III goal of < 100 mg/dL [2.6 mmol/L] and the 2003 European goal of < 2.5 mmol/L [100 mg/dL] for patients with atherosclerotic disease, type 2 diabetes, or at high risk of cardiovascular events, as assessed by a Systematic COronary Risk Evaluation [SCORE] risk ≥ 5% or < 3.0 mmol/L [115 mg/dL] for all other patients), the NCEP ATP III nonHDL-C goal (< 130 mg/dL [3.4 mmol/L]) and the combined 2003 European LDL-C and total cholesterol (TC) goals (LDL-C as stated previously and TC < 4.5 or 5.0 mmol/L [175 or 190 mg/dL] depending on risk category) at week 6[5,6]; and percentage change from baseline in HDL-C, TC, TG, nonHDL-C, lipid ratios (LDL-C/HDL-C, TC/HDL-C, and nonHDL-C/HDL-C), lipoprotein(a) (Lp [a]), apolipoprotein (Apo)A-I, ApoB, and the ApoB/ApoA-I ratio at week 6. Cost-effectiveness, frequency and severity of adverse events (AEs), and the clinical chemistry data with rosuvastatin 10 mg and atorvastatin 20 mg, were also assessed.

### Assessments

Fasting blood samples were obtained from patients at week-6 (beginning of dietary lead-in), -2, -1, 0 (randomization), and week 6, and lipid profiles analyzed at a central laboratory (Medical Research Laboratories, KY, USA for centers in the USA, Mexico, and Australia; Medical Research Laboratories, Zaventem, Belgium for European centers). Both laboratories were certified for the standardization of lipid analysis as specified by the Lipid Standardization Program of the Centers for Disease Control and Prevention, and the National Heart, Lung, and Blood Institute. Fasting concentrations of LDL-C, HDL-C, TG, TC and nonHDL-C were determined at weeks -6, -2, -1, 0 and 6, and of ApoA-I, ApoB and Lp(a) at weeks 0 and 6. Fasting LDL-C concentrations were calculated from TC, TG, and HDL-C using the Friedewald equation in patients with TG ≤ 4.5 mmol/L (400 mg/dL)[20]. A beta-quantification measurement of LDL-C was used when TG > 4.5 mmol/L (400 mg/dL). For the cost-effectiveness analysis, acquisition costs for rosuvastatin and atorvastatin within the United Kingdom and the United States were collected outside the study from pre-specified sources. US costs were the wholesale acquisition costs as reported by Medi-Span as of 6 September 2006, and UK costs were taken from the British National Formulary September 2006. Blood samples for clinical chemistry, including ALT, CK, and serum creatinine, were collected at weeks -1, 0, and 6, and AEs were recorded at weeks -2, -1, 0, and 6 by means of the standard investigator question "Have you had any health problems since the previous visit?". Patients were asked to provide a description of the event, the dates of onset and resolution, and to assess whether the AE was of mild, moderate or severe intensity. Urine samples were collected at weeks 0 and 6 for a dipstick test to measure urinary protein and blood. Study compliance was assessed at week 6 by counting returned capsules; patients were compliant if ≥ 80% of the prescribed medication was taken.

### Statistical analyses

To enable detection of a difference of 3% in mean percentage change in LDL-C between the rosuvastatin 10 mg and atorvastatin 20 mg groups with 90% power at a 5% significance level, it was estimated that 920 patients would need to be randomized using a ratio of 1:1. Allowing for an 8% withdrawal rate during the study, it was planned to randomize 1000 patients. Efficacy analyses included all patients who had a baseline lipid measurement and at least 1 post-baseline lipid measurement (intention-to-treat [ITT] population). The primary analysis used the last observation carried forward on the ITT population for patients with missing data. The ITT population was analyzed by treatment randomly allocated. Percentage changes from baseline in lipid levels at week 6 were compared by an analysis of variance with terms fitted for treatment, grouped centers, and treatment by grouped center. Goal achievement was analyzed using logistic regression analyses.

Cost-effectiveness was expressed as cost per patient achieving their NCEP ATP III or 2003 European LDL-C goals, and cost per 1% reduction in LDL-C, where costs consisted of drug acquisition costs alone. The level of efficacy achieved with 6 weeks of treatment was used to estimate the reduction that might be achieved with 1 year of continued treatment, assuming that compliance and efficacy were maintained. The analysis was carried out within both a US and a UK setting.

The safety population included all randomized patients who took at least 1 dose of study medication. No formal analysis of statistical significance was performed for the safety and tolerability data.

## Results

### Study population

**Figure 1:**
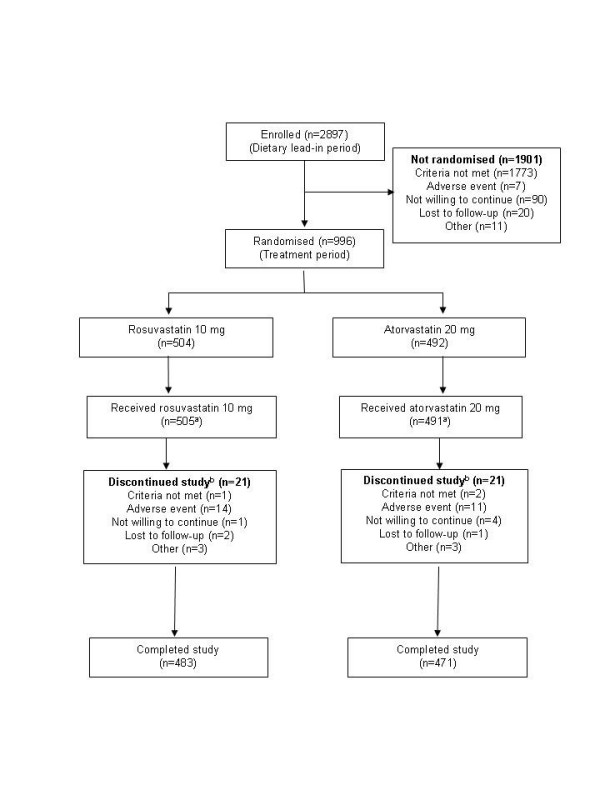
**Patient populations**. ^a^One patient randomized to atorvastatin 20 mg received rosuvastatin 10 mg. ^b^Based on intention-to-treat populations (n = 504 for rosuvastatin 10 mg; n = 492 for atorvastatin 20 mg).

Figure 1 presents the patient populations within the study and reasons for non-eligibility and discontinuations. A total of 2897 patients were enrolled in the dietary lead-in, of whom 996 were randomized to treatment (504 to rosuvastatin 10 mg, 492 to atorvastatin 20 mg); 954 patients completed the study (rosuvastatin 10 mg, n = 483; atorvastatin 20 mg, n = 471) (Figure 1). One patient was randomized to atorvastatin 20 mg and inadvertently received rosuvastatin 10 mg for the entire 6-week treatment period. As a result, the safety population comprised 505 patients in the rosuvastatin 10 mg group and 491 in the atorvastatin 20 mg group. In each treatment group, 11 patients had no post-treatment LDL-C value and therefore the ITT population was 493 for rosuvastatin 10 mg and 481 for atorvastatin 20 mg.

Baseline demographic characteristics and lipid and lipoprotein levels were similar in both treatment groups (Table 1, Table 2). More than 80% of patients had documented CHD or a CHD-risk equivalent (85.5% for rosuvastatin 10 mg, 82.7% for atorvastatin 20 mg). Of those patients without CHD or a CHD-risk equivalent, 39.5% had a Framingham 10-year risk of > 20% (Table 1).

**Table 1:** Patient demographics and baseline characteristics (randomized population)

	**Rosuvastatin 10 mg (n = 504)**	**Atorvastatin 20 mg (n = 492)**
Mean age, years (SD)	60.2 (10.4)	60.7 (10.6)
Male gender, n (%)	273 (54.2)	285 (57.9)
Mean BMI, kg/m^2 ^(SD)	29.7 (5.6)	29.7 (5.9)
Race, n (%)		
Caucasian	376 (74.6)	380 (77.2)
Hispanic	98 (19.4)	90 (18.3)
Black	23 (4.6)	17 (3.5)
Asian	6 (1.2)	3 (0.6)
Other	1 (0.2)	2 (0.4)
Renal function*, n (%)		
Normal	292 (57.9)	271 (55.1)
Mild impairment	177 (35.1)	190 (38.6)
Moderate impairment	35 (6.9)	29 (5.9)
Metabolic syndrome^†^, n (%)	254 (50.4)	237 (48.2)
Diabetes (type 1 or 2), n (%)	256 (50.8)	250 (50.8)
CHD or CHD-risk equivalents, n (%)	431 (85.5)	407 (82.7)
Patients without CHD or a CHD-risk equivalent, n (%)	72 (14.3)	85 (17.3)
Framingham 10-year risk > 20%	30 (6.0)	32 (6.5)
Framingham 10-year risk ≥ 10% and ≤ 20%	17 (3.4)	25 (5.1)
Framingham 10-year risk < 10%	25 (5.0)	28 (5.7)

SD, standard deviation; BMI, body mass index; CHD, coronary heart disease; NCEP ATP, National Cholesterol Education Program Adult Treatment Panel; TG, triglycerides; HDL-C, high-density lipoprotein cholesterol; SBP, systolic blood pressure; DBP, diastolic blood pressure.

*Renal function assessed using creatinine clearance as follows: > 80 mL/min (normal), 50 to ≤ 80 mL/min (mild impairment), 30 to < 50 mL/min (moderate impairment). Creatinine clearance was calculated from serum creatinine using the following equations: creatinine clearance (mL/min) = ([140-age at visit 4] × weight at visit 1 [kg])/72 × serum creatinine at visit 4 (mmol/L) × 0.01131 if male; or creatinine clearance (mL/min) = ([140-age at visit 4] × weight at visit 1 [kg])/85 × serum creatinine at visit 4 (mmol/L) × 0.01131 if female.

^†^Modified NCEP ATP III definition [6]: Patients fulfill at least 3 of the following 5 criteria: fasting blood glucose ≥ 6.1 and ≤ 6.9 mmol/L (110 and 125 mg/dL); waist circumference > 102 cm (male) or > 88 cm (female); TG ≥ 1.7 mmol/L (150 mg/dL); HDL-C < 1.0 mmol/L (40 mg/dL) in men or < 1.3 mmol/L (50 mg/dL) in women; and hypertension (SBP ≥ 130 mmHg or DBP ≥ 85 mmHg) or taking antihypertensive medication.

**Table 2:** Change from baseline in lipoprotein and lipid levels after 6 weeks of treatment (ITT population)

Lipids/lipoproteins	Rosuvastatin 10 mg (n = 493)		Atorvastatin 20 mg (n = 481)		p value*
		
	Mean baseline level, mg/dL	LSM percentage change (SE)	Mean baseline level, mg/dL	LSM percentage change (SE)	
LDL-C	165.1	-44.6 (0.6)	164.9	-42.7 (0.6)	< 0.05
TC	250.9	-30.8 (0.5)	250.9	-30.7 (0.5)	ns
HDL-C	50.3	6.4 (0.5)	49.9	3.1 (0.5)	< 0.001
TG	178.1	-17.9 (1.2)	180.3	-19.1 (1.2)	ns
NonHDL-C	200.6	-40.1 (0.6)	200.9	-38.9 (0.6)	ns
LDL-C/HDL-C	3.5	-47.6 (0.7)	3.5	-44.0 (0.7)	< 0.001
TC/HDL-C	5.3	-34.6 (0.5)	5.3	-32.3 (0.5)	< 0.01
NonHDL-C/HDL-C	4.3	-43.3 (0.6)	4.3	-40.2 (0.7)	< 0.001
Lp(a)	32.6	2.1 (3.8)	27.0	13.3 (3.8)	< 0.05
ApoB	157.4	-35.2 (0.6)	156.6	-34.1 (0.6)	ns
ApoA-I	160.5	4.8 (0.5)	159.6	1.7 (0.5)	< 0.001
ApoB/ApoA-I	1.0	-37.6 (0.7)	1.0	-34.6 (0.7)	0.001

ITT, intention to treat; LSM, least-squares mean; SE, standard error of the mean; LDL-C, low-density lipoprotein cholesterol; TC, total cholesterol; HDL-C, high-density lipoprotein cholesterol; TG, triglycerides; Lp, lipoprotein; Apo, apolipoprotein.

To convert cholesterol inmg/dL tommol/L, multiply by .02586; to convert TG in mg/dL to mmol/L, multiply by .01129.

*p value obtained from analysis of variance comparing rosuvastatin 10 mg with atorvastatin for LSM percentage change in lipid and lipoproteins.

On completion of the study, 90.1% of patients in the rosuvastatin 10 mg group and 89.6% of patients in the atorvastatin 20 mg group were compliant with study medication.

### Efficacy

#### LDL-C

Rosuvastatin 10 mg was significantly more effective at reducing the primary efficacy variable, LDL-C level, than atorvastatin 20 mg after 6 weeks of treatment (mean change -44.6% vs. -42.7%, p < 0.05) (Table 2).

#### Lipid goals – LDL-C

In terms of secondary efficacy variables, rosuvastatin 10 mg enabled more patients to achieve both the NCEP ATP III and the 2003 European LDL-C goals than atorvastatin 20 mg (68.8% vs. 62.5%, p < 0.05; 68.0% vs. 63.3%, p < 0.05, respectively). Furthermore, the 2003 European LDL-C goal was achieved by 65.6% of patients at greatest risk (established CVD, type 2 diabetes, LDL-C ≥ 6 mmol/L, TC ≥ 8 mmol/L or blood pressure ≥ 180/110 mmHg) receiving rosuvastatin 10 mg and by 60.3% of at greatest-risk patients receiving atorvastatin 20 mg.

#### Lipid goals – nonHDL-C and combined goals

The NCEP ATP III nonHDL-C goal of < 130 mg/dL (3.4 mmol/L) was achieved by 69.7% of patients receiving rosuvastatin 10 mg and 65.0% of patients receiving atorvastatin 20 mg (p = ns). In patients with TG ≥ 2.3 mmol/L (200 mg/dL) (n = 292), 62.1% of those receiving rosuvastatin 10 mg achieved the combined NCEP ATP III LDL-C and nonHDL-C goals at week 6, compared with 55.8% of those receiving atorvastatin 20 mg. The proportion of patients achieving combined 2003 European LDL-C and TC goals was 55.2% for rosuvastatin 10 mg and 53.3% for atorvastatin 20 mg (p = ns).

#### Improvements across the lipid profile

Rosuvastatin 10 mg increased HDL-C levels to a significantly greater extent than atorvastatin 20 mg (mean change 6.4% and 3.1%, p < 0.001), while similar reductions in TC, TG, and nonHDL-C levels were observed with both treatments (Table 2). Ratios of LDL-C/HDL-C, TC/HDL-C, and nonHDL-C/HDL-C were all reduced to a significantly greater extent with rosuvastatin 10 mg than with atorvastatin 20 mg (p < 0.001, p < 0.01, p < 0.001, respectively) (Table 2). Lp(a) was increased by 2.1% for rosuvastatin 10 mg and 13.3% for atorvastatin 20 mg (p < 0.05). Greater improvements in ApoA-I and the ApoB/ApoA-I ratio were also seen in the rosuvastatin 10 mg group compared with the atorvastatin 20 mg group (p < 0.001, p = 0.001, respectively), while reductions in ApoB were similar between treatments (Table 2).

### Cost-effectiveness

The yearly acquisition costs were lower for rosuvastatin 10 mg compared with atorvastatin 20 mg in both the US ($959.95 vs. $1204.50) and the UK (£235.03 vs. £321.20). Furthermore, rosuvastatin 10 mg is more effective at a lower cost than atorvastatin 20 mg both in terms of LDL-C reduction and patients achieving NCEP ATP III or 2003 European LDL-C goals.

### Safety and tolerability

Both treatments were well tolerated and the overall frequency and type of AEs were similar between treatment groups (Table 3). An AE was experienced by 27.5% patients receiving rosuvastatin 10 mg and 26.1% of those receiving atorvastatin 20 mg; most were of mild-to-moderate intensity (94.2% and 96.1%, respectively) and a small number were classified as severe (5.8% and 3.9%, respectively). Myalgia and urinary tract infections were the most frequently reported AEs in both treatment groups (Table 3). The other most frequently reported AEs included headaches and nausea.

**Table 3:** Most frequent (≥ 1.0%) treatment-emergent adverse events (randomized safety population) from the open-label PULSAR trial

	Number (%) of patients with adverse events	
	
	Rosuvastatin 10 mg (n = 505)	Atorvastatin 20 mg (n = 491)
Any adverse event	139 (27.5)	128 (26.1)
Myalgia	24 (4.8)	13 (2.6)
Urinary tract infection	13 (2.6)	16 (3.3)
Headache	8 (1.6)	7 (1.4)
Nausea	4 (0.8)	9 (1.8)
Bone pain	8 (1.6)	3 (0.6)
Muscle cramp	5 (1.0)	3 (0.6)
Peripheral edema	3 (0.6)	5 (1.0)

A small number of patients discontinued treatment as a result of an AE or a drug-related AE (2.8% and 2.4% for rosuvastatin 10 mg, 2.2% and 2.0% for atorvastatin 20 mg). The most common reported AEs leading to discontinuation (occurring in > 1 patient in either treatment group) were myalgia, asthenia, bone pain, headache, and muscular weakness. Few SAEs were reported (1.4% for rosuvastatin 10 mg, 1.2% for atorvastatin 20 mg) and no drug-related SAEs were recorded with either treatment. Two deaths occurred during the study (both in the rosuvastatin 10 mg group, one resulting from cardiac failure and one from MI) and neither of these was considered related to study treatment and both could be expected in this type of study population.

Of the 24 (4.8%) treatment-emergent myalgia cases in the rosuvastatin 10 mg group and the 13(2.6%) cases in the atorvastatin 20 mg group, 13 (2.6%) and 7 (1.4%) were thought to be treatment-related, and 4 (0.8%) and 1 (0.2%) cases resulted in withdrawal from the study. None of the 37 patients with reported myalgia had clinically important elevations in CK levels (> 10 × ULN) or even CK > 3 × ULN. One additional patient receiving rosuvastatin 10 mg had an increase in CK of > 5 × ULN, but this was not associated with muscle symptoms and not considered clinically important. The mean percentage change in CK from baseline was 18.6% in the rosuvastatin 10 mg group and 14.9% in the atorvastatin 20 mg group. Changes from baseline to the end of treatment for all serum chemistry measurements were small and similar between the two treatments. Clinically important elevations in ALT (> 3 × ULN on two consecutive occasions) were recorded for one patient receiving atorvastatin 20 mg, although this was not considered to be drug-related. In addition, no patients showed an increase in serum creatinine > 50% from baseline and > ULN with either treatment.

There were no clinically important abnormalities in urinalysis and no treatment-related trends were reported, with a low incidence of proteinuria (increase in dipstick-positive urine protein from "none" or "trace" at baseline to "≥ ++" at week 6) (0.6% in both groups) and hematuria (increase in dipstick-positive urine blood from "none" or "trace" at baseline to "≥ +" at week 6) with rosuvastatin 10 mg and atorvastatin 20 mg (2.6% vs. 2.2%). No cases of rhabdomyolysis, myopathy, or acute renal failure were observed.

## Discussion

Although a number of previous studies have compared atorvastatin with rosuvastatin in patients with hypercholesterolemia, some did not include high-risk patients, some were local studies conducted in a single country, and many were not powered to study efficacy in terms of LDL-C lowering [21-24]. PULSAR is the first, prospective, large-scale, multinational study designed to compare low doses of rosuvastatin and atorvastatin for their LDL-C-lowering efficacy in high-risk patients. The PULSAR study is part of a wider program investigating the efficacy and safety of rosuvastatin[25]. The program was designed to address the hypothesis that the statin with the greatest efficacy for improving the atherogenic lipid profile and beneficially modifying inflammatory markers will also slow progression of atherosclerotic plaques, and consequently, result in the greatest reductions in cardiovascular morbidity and mortality[25]. PULSAR is one of the studies designed to address the first part of the hypothesis, investigating the effects of rosuvastatin on the lipid profile. The results of the PULSAR study demonstrate that rosuvastatin 10 mg was significantly more effective than atorvastatin 20 mg at reducing LDL-C levels in high-risk patients with hypercholesterolemia.

This is consistent with findings from previous studies that have compared rosuvastatin 10 mg and atorvastatin 20 mg in patients with hypercholesterolemia. In three separate studies, one with 2431 patients with hypercholesterolemia (LDL-C ≥ 160 and < 250 mg/dL [4.1 and 6.5 mmol/L]), one with 461 patients (aged 40–80 years) with CHD and low HDL-C, and one with 263 patients with type 2 diabetes, rosuvastatin 10 mg was more effective at reducing LDL-C than atorvastatin 20 mg after 6 weeks of treatment (45.8% vs. 42.6%, 44.0% vs. 38.4%, 45.9% vs. 41.3%, respectively; all p < 0.05)[11,26,27]. Furthermore, in an 8-week study of 3140 high-risk patients with hypercholesterolemia and CHD, atherosclerosis, type 2 diabetes, or a 10-year CHD risk > 20%, rosuvastatin 10 mg was also significantly more efficacious than atorvastatin 20 mg at reducing LDL-C (47.0% vs. 43.7%, p < 0.001)[12].

Consistent with the greater LDL-C-lowering efficacy in the PULSAR study, more patients treated with rosuvastatin 10 mg achieved recommended 2003 European and NCEP ATP III LDL-C goals than with atorvastatin 20 mg. Furthermore, a greater proportion of patients at highest risk (with established CVD, type 2 diabetes, LDL-C ≥ 6 mmol/L, TC ≥ 8 mmol/L, or blood pressure ≥ 180/110 mmHg) achieved the more stringent European LDL-C goal of < 2.5 mmol/L (100 mg/dL) with rosuvastatin 10 mg than with atorvastatin 20 mg. A previous study of 2829 high-risk patients showed that 52% did not achieve a LDL-C goal of < 2.5 mmol/L (100 mg/dL) with the initial statin dose[9]. Of the remaining patients, 55% did not have their dosage up-titrated, and of those whose treatment was titrated to a higher dose, only 31% achieved the LDL-C goal. Therefore, selecting a statin that is more efficacious at starting dose will reduce the need for dose titration and improve goal achievement, potentially leading to benefits in CVD-risk reduction.

HDL-C is thought to have a protective role against the development of atherosclerotic plaques[28] and a low HDL-C level is considered a risk factor for CHD. Agents that improve HDL-C as well as lower LDL-C may offer additional benefits for CHD-risk reduction. In the present study, increases in HDL-C were significantly greater with rosuvastatin 10 mg than with atorvastatin 20 mg.

Several studies have shown that other lipid parameters, such as apolipoproteins and lipid ratios, are better predictors of CVD risk than LDL-C and may be used to guide therapeutic decisions[29]. For example, in the AMORIS study of 175,553 individuals, ApoB was found to be a stronger predictor than LDL-C for risk of fatal MI[30]. Furthermore, the ApoB/ApoA-I ratio was found to be the most effective predictor of MI in the INTERHEART study of 15,152 patients with CHD from 52 countries[31]. In the present study, patients receiving rosuvastatin 10 mg also showed greater improvements in levels of ApoA-I, and ratios of LDL-C/HDL-C, TC/LDL-C, nonHDL-C/HDL-C, and ApoB/ApoA-I, compared with those receiving atorvastatin 20 mg. Reductions in TC, TG, nonHDL-C, and ApoB levels were similar between treatments. Thus, the results of the PULSAR study are consistent with those from previous studies comparing the effects of rosuvastatin 10 mg and atorvastatin 20 mg on lipid parameters. Rosuvastatin 10 mg significantly improved levels of HDL-C, TC, and nonHDL-C and ratios of LDL-C/HDL-C and TC/HDL-C, compared with atorvastatin 20 mg in previous studies with high-risk patients[12,26,27]. Improvements in the atherogenic lipid profile may be beneficial for reducing global risk in patients with CHD[6]. Furthermore, as part of the wider rosuvastatin clinical trial program, one study has reported that rosuvastatin 40 mg can arrest and even reverse progression of atherosclerosis, in association with reductions in LDL-C[32]. Ultimately, to assure the clinical relevance of changes in the lipid profile, the greater efficacy of rosuvastatin in terms of LDL-C lowering must translate into reductions in morbidity and mortality. As such, several outcomes studies are now underway to assess the efficacy of rosuvastatin in high-risk patients [33-35].

Results of the PULSAR study showed rosuvastatin 10 mg to be a cost-effective alternative to atorvastatin 20 mg, both in terms of cost per percentage LDL-C reduction and cost per patient achieving their NCEP ATP III or 2003 European LDL-C goal, both in a UK and US setting. These results are in line with several previous cost-effectiveness analyses, which reported rosuvastatin to be more cost-effective than atorvastatin, pravastatin and simvastatin [36-38]. A recent UK study showed simvastatin 40 mg to be a cost-effective alternative to atorvastatin 20 mg, and has stressed the importance of comparing costs when considering statin therapies[39]. The study reported the benefits of switching to more cost-effective statins in terms of potential savings to the health service[39]. Further economic analyses of rosuvastatin are now needed to determine its potential as a more cost-effective therapy compared with other statins.

Both rosuvastatin and atorvastatin were well tolerated in this study, and none of the reported AEs were unexpected given the age and underlying medical conditions of the patient population. Most AEs were of mild or moderate severity, and were not considered to be treatment-related. The most commonly reported AE was myalgia, although none of the cases were associated with a clinically important elevation in CK (> 10 × ULN) (or even a CK > 3 × ULN). Furthermore, there were no reports of rhabdomyolysis, renal, or liver insufficiencies during the study. It is possible that the open-label design of the study could potentially have biased reporting of AEs, especially since the study was conducted at a time of high media activity related to rosuvastatin. This is supported by an NLA analysis of AE reporting rates of several statins, which found that the reporting of rosuvastatin-associated rhabdomyolysis and renal failure increased following media publicity[19]. However, the AE reporting patterns of rosuvastatin did not differ greatly from those of other statins, once differential reporting effects were taken into account.

Results from the PULSAR study are consistent with previous studies, which have assessed the safety of rosuvastatin and atorvastatin in a range of patients with dyslipidemia[16,40]. In a global analysis of 12,400 patients in the rosuvastatin phase II/III clinical program, Shepherd et al[16] found that rosuvastatin 5–40 mg had a similar safety profile to other statins, and demonstrated a favorable benefit-risk profile across this dose range. Nevertheless, there has been some concern regarding the potential toxicity of rosuvastatin, particularly in terms of renal and muscle events [41]. However, the Food and Drug Administration recently conducted a comprehensive review of available safety data from pre-clinical studies, pre-marketing clinical trials, phase IV studies and post-marketing AE reports [42]. The review concluded that rosuvastatin poses no greater risk of muscle toxicity or serious renal injury compared with other statins [42]. Furthermore, a pooled data analysis of 9416 patients in 44 clinical trials found that the safety of atorvastatin 10–80 mg was also similar to that of other statins[40]. Results from 2 recent large-scale, independent, pharmacoepidemiologic studies conducted in The Netherlands and the USA were in accordance with these findings [17,18]. These studies of real-world patient data in over 96,000 patients receiving statin therapy found that the incidence of pre-defined events requiring hospitalization associated with the muscle, liver or kidneys was the same for all currently marketed statins [17,18].

## Conclusion

In conclusion, at recommended starting doses, rosuvastatin (10 mg) was more efficacious than atorvastatin (20 mg), in terms of LDL-C lowering, LDL-C goal achievement, and improving the atherogenic lipid profile. The greater efficacy of rosuvastatin at starting dose should help to reduce the need for dose titration and enable more patients to achieve recommended treatment goals in clinical practice. In addition, improvements across the whole atherogenic lipid profile, including increases in HDL-C, may provide further reductions in the risk of CVD.
